# Computational Simulation of the Electronic State Transition in the Ternary Hexagonal Compound BaAgBi

**DOI:** 10.3389/fchem.2021.796323

**Published:** 2021-11-11

**Authors:** Yu Chang, Xin Wang, Sanggyun Na, Weiwei Zhang

**Affiliations:** ^1^ Tonghua Normal University, Tonghua, China; ^2^ Wonkwang University, Iksan, South Korea

**Keywords:** first-principles calculation, electronic band structure, topological nodal line, DFT, ternary hexagonal compound

## Abstract

Topological properties in metals or semimetals have sparked tremendous scientific interest in quantum chemistry because of their exotic surface state behavior. The current research focus is still on discovering ideal topological metal material candidates. We propose a ternary compound with a hexagonal crystal structure, BaAgBi, which was discovered to exhibit two Weyl nodal ring states around the Fermi energy level without the spin–orbit coupling (SOC) effect using theoretical calculations. When the SOC effect is considered, the topological phases transform into two Dirac nodal line states, and their locations also shift from the Weyl nodal rings. The surface states of both the Weyl nodal ring and Dirac nodal lines were calculated on the (001) surface projection using a tight-binding Hamiltonian, and clear drumhead states were observed, with large spatial distribution areas and wide energy variation ranges. These topological features in BaAgBi can be very beneficial for experimental detection, inspiring further experimental investigation.

## Introduction

Since the discovery of topological insulators, the study of topological properties in materials has sparked extremely large research attention in material science, particularly in solid-state physics and chemistry (Bradlyn et al., 2017; Yan and Felser, 2017; Schoop et al., 2018). With the ongoing development, the current research into topological materials has been expanded into metals or semimetals (Burkov, 2016; Yan and Felser, 2017; Yu et al., 2017; Zhang et al., 2019a; Gao et al., 2019). Contrary to that in conventional topological insulators, the topological states in metals are characterized by linear band crossings in the low-energy region around the Fermi level, and they are protected by structural symmetry and nontrivial band topology. Topological states in metals can be classified into different types based on different band crossing conditions and intertwining shapes. For example, nodal point (Zhang et al., 2017a; Cano et al., 2019; He et al., 2019; Li et al., 2020; Li and Xia, 2020), nodal line (Chang et al., 2016a; Hosen et al., 2018; Kim et al., 2018; Takane et al., 2018; Zheng et al., 2019; Wang et al., 2020a; Wang et al., 2020b; He et al., 2020; Jin et al., 2020; Wang et al., 2021a; He et al., 2021; Zhou et al., 2021), and nodal surface (Fu et al., 2019; Yang et al., 2019; Yang et al., 2020; Yang and Zhang, 2020) can be differentiated by their band crossing dimensionality: Weyl (Huang et al., 2015; Soluyanov et al., 2015; Chang et al., 2016b; Jia et al., 2016; Wang et al., 2018a), triple (Jin et al., 2019a; Bhattacharya et al., 2021), Dirac (Galanakis and Mavropoulos, 2007; Heikkilä and Volovik, 2011; He et al., 2016; Zhang et al., 2017b; Zhang et al., 2018a; Wang et al., 2018b; Wang et al., 2019), sextuple, and octuple topological states (Bradlyn et al., 2016), which can also be distinguished by their band crossing degeneracy. Some other classifications can also be defined based on their band dispersion rates or band crossing shapes (Bzdušek et al., 2016; Chen et al., 2017; Wang et al., 2017; Zhang et al., 2018b).

For topological nodal points or nodal lines, their linear band crossings are often associated with protected surface states (Zhang et al., 2017a; Zhang et al., 2017b; Chen et al., 2017; Sheng et al., 2017; Jin et al., 2019b; Zhang et al., 2019b; He et al., 2019; Liu et al., 2019; Wang et al., 2020c; Wang et al., 2020d; Wang et al., 2020e; Meng et al., 2020; Yang et al., 2021), i.e., Fermi arc states connecting the nodal points and drumhead surface states concatenating the nodal lines. The nodal line can be regarded as a link between innumerous nodal points, and the corresponding drumhead surface state is a union of infinite Fermi arc states. In this regard, studying nodal line metals or even employing them for future applications is advantageous simply because it could provide more possibilities and varieties. The current research focus is on discovering nodal line metals with clean band structures, and more topological metal materials are being discovered and even designed as the theoretical calculation tools and computation power improve. Some of them have also been successfully verified through experimental characterizations (Jia et al., 2016; Du et al., 2017; Wang et al., 2017; Hosen et al., 2018; Kim et al., 2018; Takane et al., 2018; Fu et al., 2019; Wang et al., 2021a). However, the number of ideal topological metals is still very limited even with high-throughput computation methods.

Herein, we present BaAgBi, a ternary compound with a hexagonal structure. When the spin–orbit coupling (SOC) effect is not considered, its metallic band structures exhibit multiple band crossing points near the Fermi level, which correspond to two Weyl nodal ring states, according to the first-principles calculations. The detailed energy variation and spatial distribution of the nodal rings are examined using a three-dimensional band dispersion scan. When the SOC effect is considered, the original Weyl nodal rings are gapped out, and new Dirac nodal line states emerge, with their locations shifted as well. The corresponding surface states for both the Weyl nodal rings and Dirac nodal lines were calculated by constructing a tight-binding Hamiltonian and a surface slab model, and clear drumhead states were discovered along the (001) surface projection spectrum. This BaAgBi material can serve as an ideal nodal line metal for studying the related exotic physical properties since these surface states with large energy variations and wide spatial distributions can be very useful for experimental detection.

## Computational Methodology

We used the Vienna ab initio simulation package (VASP) (Hafner, 2008) to perform the first-principles calculations to examine the electronic band structures of the BaAgBi material. Under the density functional theory (Payne et al., 1992), the generalized gradient approximation (Perdew et al., 1996) of the Perdew–Burke–Ernzerhof (PBE) functional (Ernzerhof and Scuseria, 1999) was used to determine the correlation exchange potential. A cutoff energy of 500 eV was selected for the plane wave set, and a Monkhorst–Pack k-mesh of 9 × 9 × 5 was used for the first Brillouin zone sampling. The structure was fully relaxed when the total force per atom was less than 1 × 10^–3^ eV/Å, and the energy convergence was reached when the total energy difference per atom was smaller than 5 × 10^–6^ eV. The open-source VASPKIT package (Wang et al., 2021b) was used to extract the calculation data. Maximally localized Wannier functions were constructed with the Wannier90 (Mostofi et al., 2008; Mostofi et al., 2014) code to investigate the topological properties, and based on them, the surface states were calculated using the WANNIERTOOLS package (Wu et al., 2018).

## Results and Discussions

**FIGURE 1:**
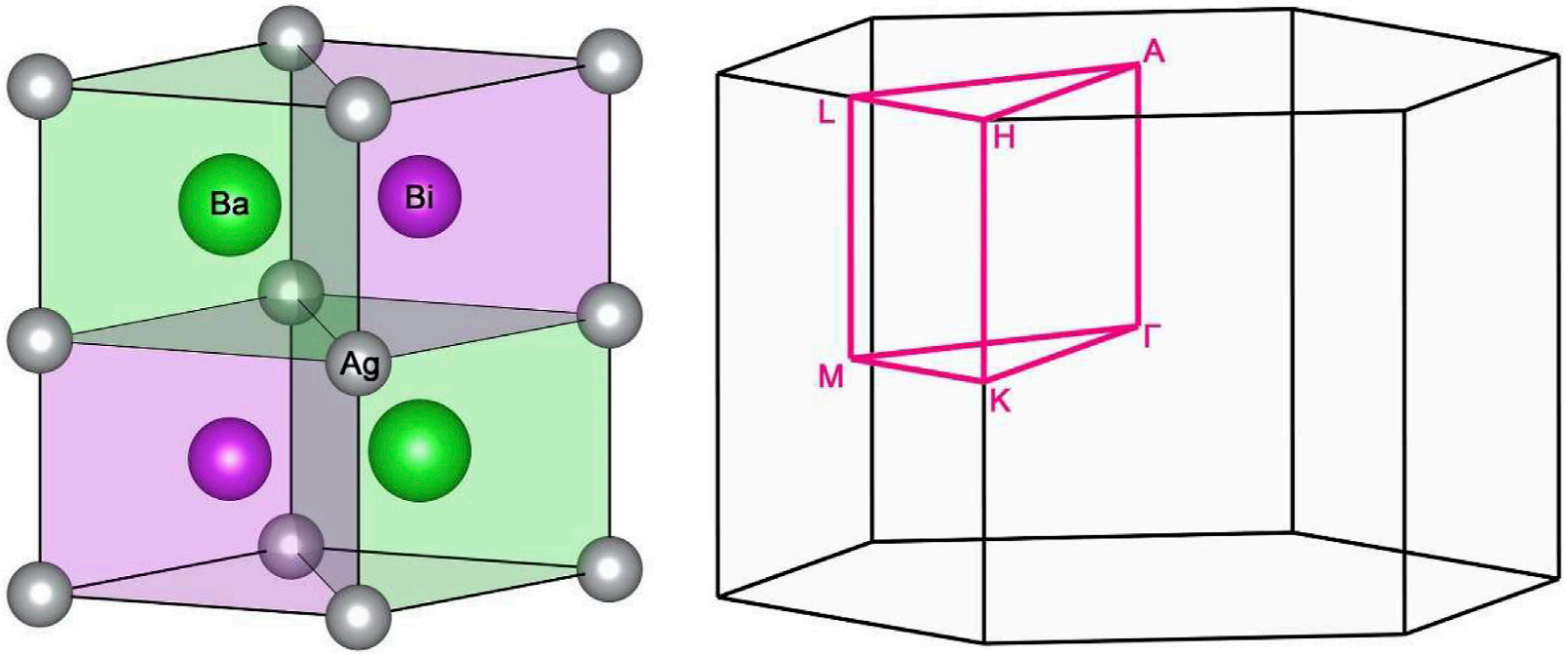
**(A)** The crystal structure of the BaAgBi material and **(B)** its corresponding Brillouin zone, with high symmetry points and paths marked.

The ternary compound, BaAgBi, has a hexagonal structure with space group P6_3_/mmc (No. 194). As shown in Figure 1A, the unit cell of BaAgBi contains two Ag atoms at the 2a Wyckoff sites (0, 0, 0), two Ba atoms at the 2c Wyckoff sites (1/3, 2/3, 1/4), and two Bi atoms at the 2d Wyckoff sites (1/3, 2/3, 3/4). The calculated lattice constants are a = b = 5.823 Å and c = 7.040 Å, which are used in the electronic band structure analysis. Before we proceed, it is necessary to note that the current crystal has two symmetry operations: spatial inversion symmetry Ψ and time-reversal symmetry Τ, which are very important for the investigation of its topological properties.

**FIGURE 2:**
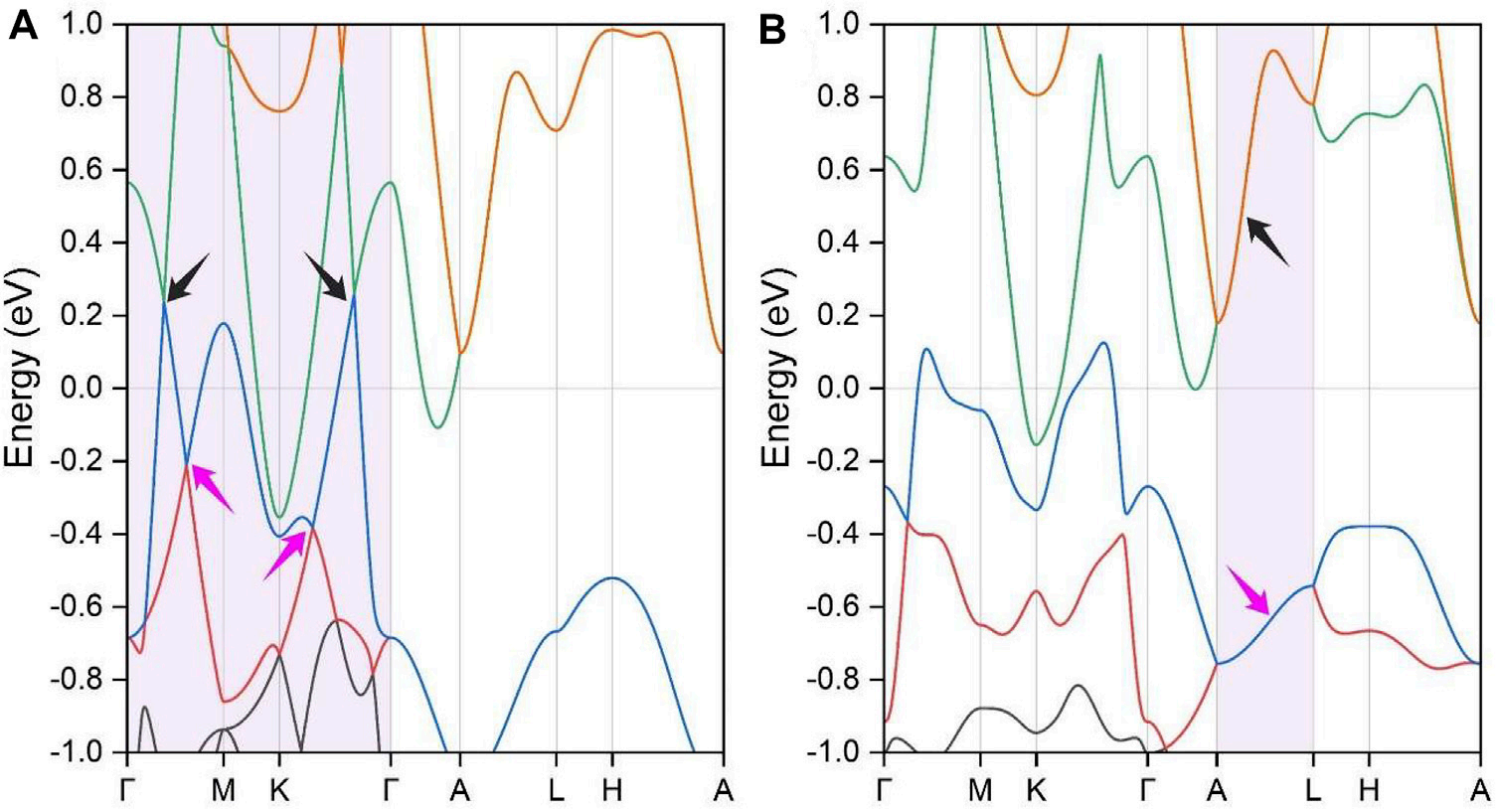
The calculated electronic band structures of the BaAgBi material **(A)** without the SOC effect and **(B)** with the SOC effect. The topological band crossing areas are indicated by the arrows. In this figure, each band is highlighted by different colors.

**FIGURE 3:**
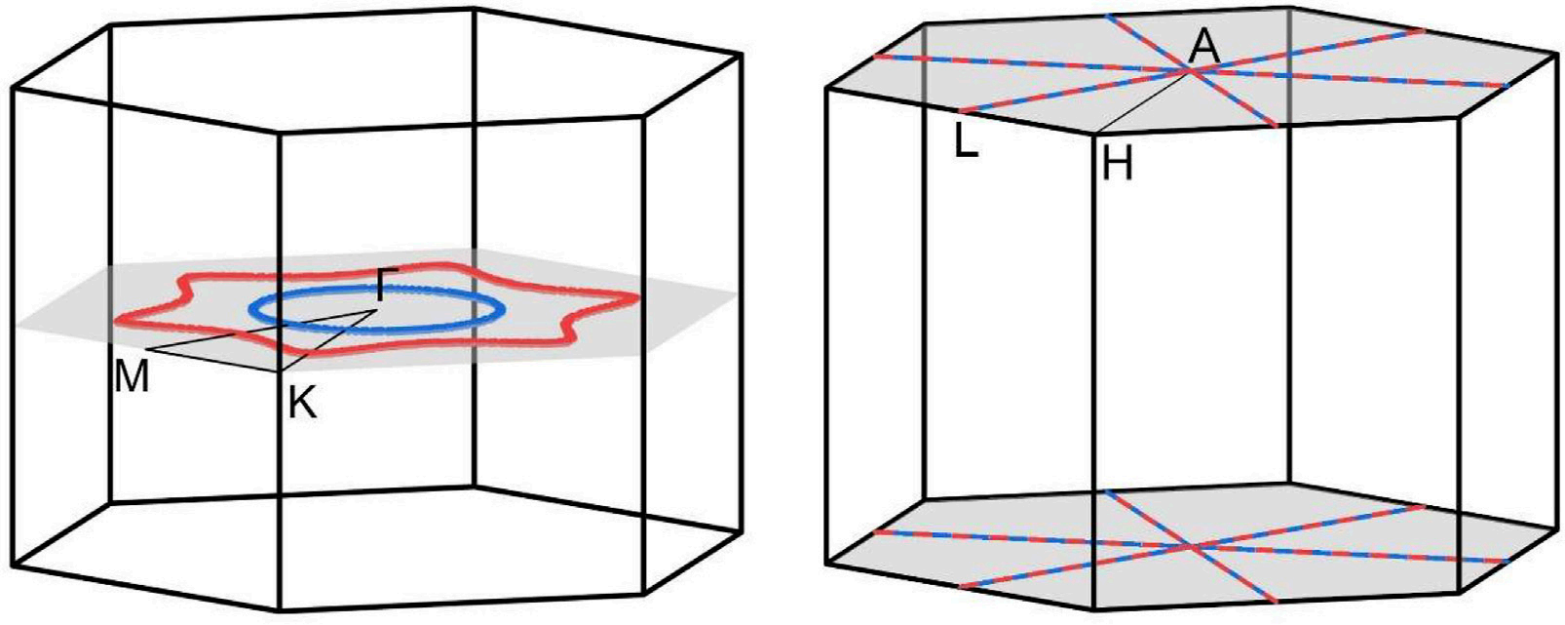
The illustration of **(A)** the location and distribution of the two Weyl nodal rings along the k_z_ = 0 plane and **(B)** the two Dirac nodal lines along the k_z_ = π the plane. The high symmetry points and paths are also displayed for enhanced visualization.

The electronic band structures of BaAgBi were calculated, and the results are shown in Figure 2A. Notably, the Fermi energy level is shifted to 0 eV in the figure, and only the bands near the Fermi level are shown. The SeeK-path tool was used to select the k paths, and their location in the first Brillouin zone is shown in Figure 1B. There are several bands across the Fermi energy, indicating that the BaAgBi compound has a metallic feature. Furthermore, these bands exhibit multiple crossing points around the Fermi level, as labeled in the figure. A closer examination reveals that these band crossings belong to two pairs formed from three bands that are red, green, and blue. Overall, these band crossings points are located along two high symmetry paths, Γ–M and K–Γ. As shown in Figure 1B, these two paths belong to the same plane of k_z_ = 0 at the Γ point. Since the BaAgBi crystal has both spatial inversion symmetry and time-reversal symmetry, these crossing points should not be isolated but should belong to the nodal rings. A precise band dispersion scan was performed along the whole plane of k_z_ = 0, and the results are reported in the supplementary materials. According to the results, these three bands did form two nodal rings, which are centered at the Γ point. Figure 3A shows the location and distribution of the two Weyl nodal rings. The big red nodal ring has a rounded hexagram shape, whereas the small blue nodal ring has a circle shape. The bigger nodal ring has a much larger energy variation than the smaller one. They are both classified as Type-I because of the reverse band dispersion rates around them.

Since the BaAgBi material contains heavy metal elements, the SOC effect should be considered. Thus, we calculated the band structures under the SOC effect, and the results are displayed in Figure 2B. Note that every band in Figure 2 is doubly degenerate. When the SOC effect is neglected, it can be observed that the original nodal ring band crossing states along the Γ–M and K–Γ paths are completed destroyed. The inclusion of the SOC effect is well known to gap out topological band crossings, and this behavior is particularly noticeable in the current material because of the presence of all three heavy metal elements. However, as indicated by the arrows in the figure, two new band crossing lines emerge, forming two Dirac nodal lines along the A–L path in the k_z_ = π plane. Based on the rotation symmetry of the BaAgBi compound, there should be two more pairs of the same Dirac nodal lines in the same plane, all of which are symmetrically equivalent. A precise band dispersion scan was also performed, and the results are reported in the supplementary materials, from which these Dirac nodal lines can be clearly observed. Under the SOC effect, the two Weyl nodal ring states transform into two Dirac nodal line states, and their locations also shift from the k_z_ = 0 plane to the k_z_ = π plane. Figure 3B shows a schematic illustration of the location and distribution of the two Dirac nodal lines, which have hexagonal star lines.

**FIGURE 4:**
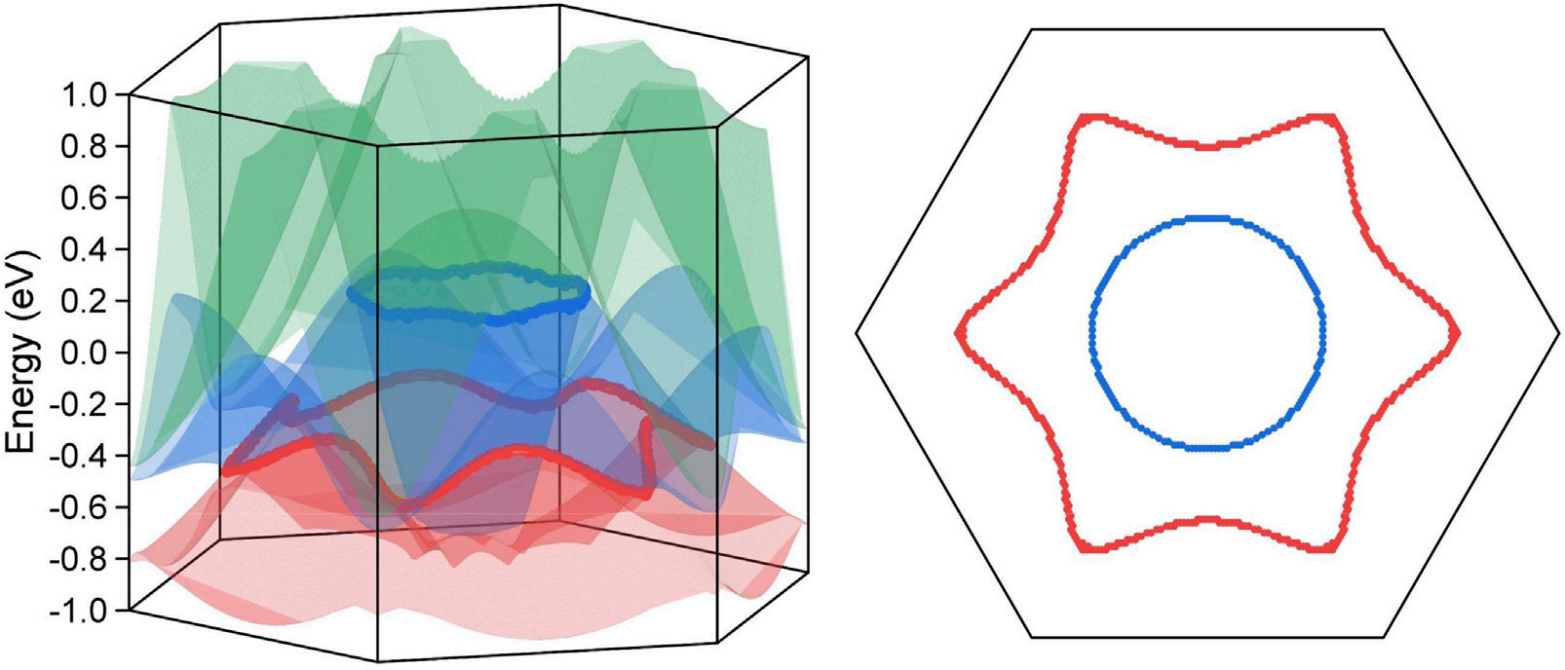
The three dimensional surface dispersion of the three crossing bands along the k_z_ = 0 plane for BaAgBi without SOC effect in the left panel. The crossing lines are labelled by red and blue dots, which correspond to the two Weyl nodal rings. The exact spatial distribution of the two Wey nodal rings along the k_z_ = 0 plane in the right panel.

**FIGURE 5:**
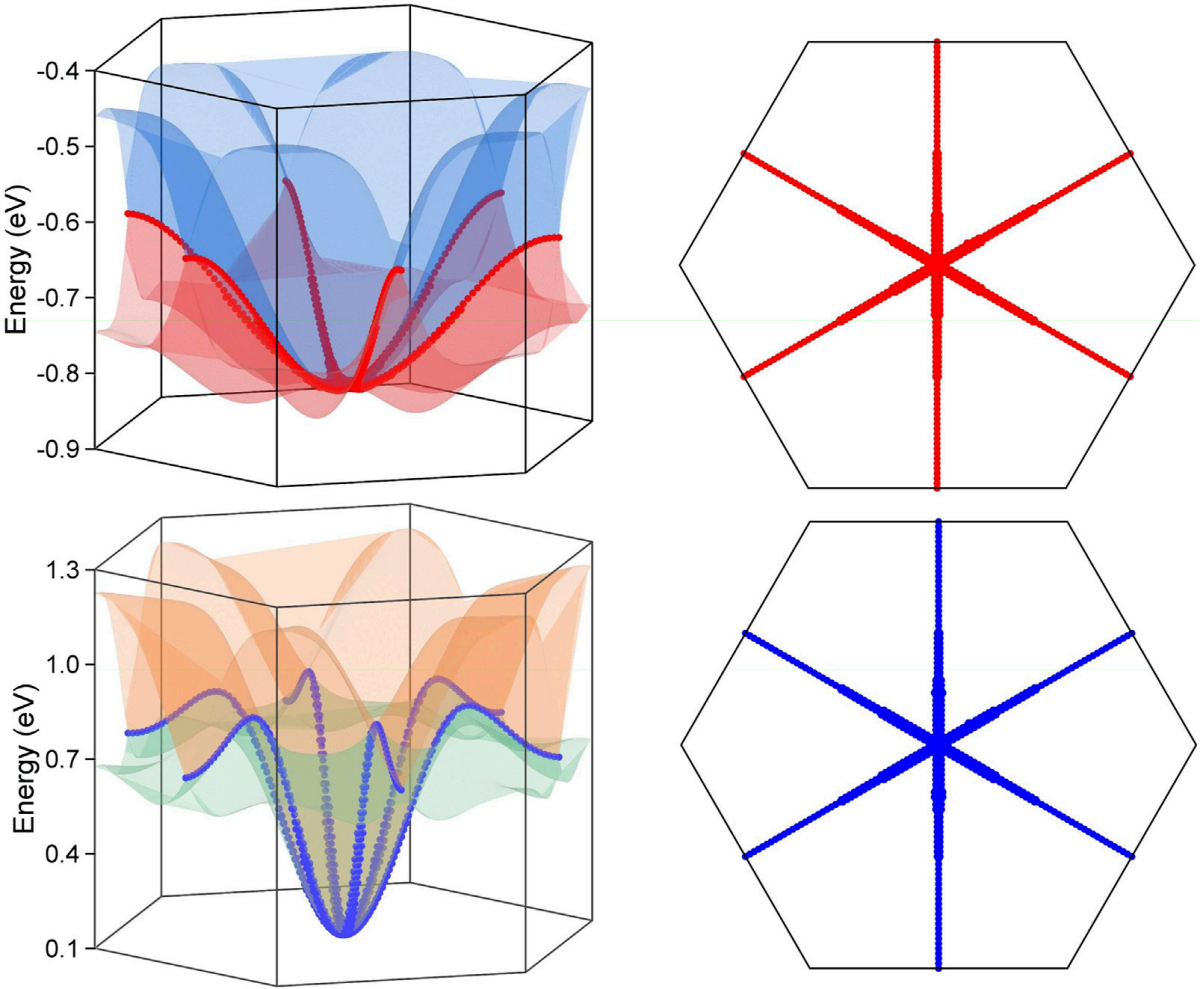
The three dimensional surface dispersion of the crossing bands along the k_z_ = π plane for BaAgBi with SOC effect in the left panel. The crossing lines are labelled by red and blue dots, which correspond to the two Dirac nodal lines. The exact spatial distribution of the two Dirac nodal rings along the k_z_ = π plane in the right panel.

The three dimensional band dispersion has been scanned along the k_z_ = 0 plane for the two Weyl nodal rings and along the k_z_ = π plane for the Dirac nodal lines, and the calculation results are reported in Figures 4, 5, respectively. The crossing lines are marked by the red and blue dot in the figure and their exact spatial location are shown in the right panel of each figure.

**FIGURE 6:**
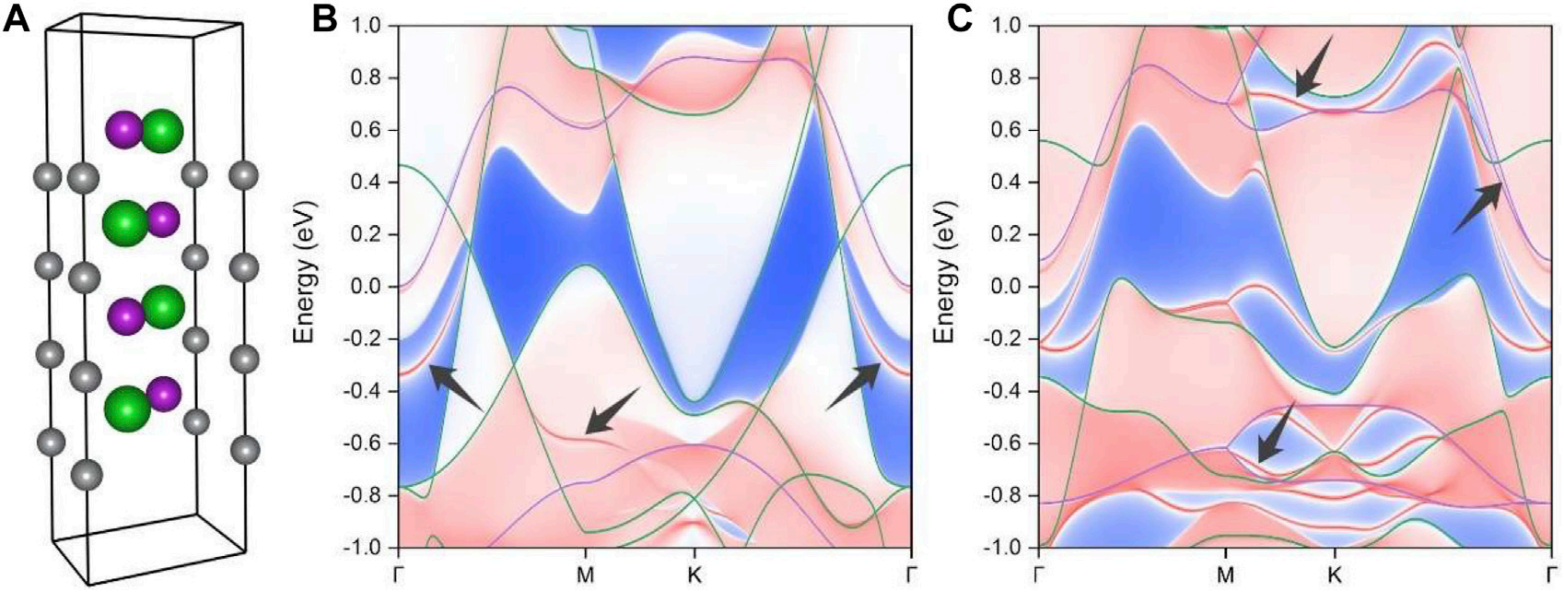
**(A)** The slab model along the (001) surface with only a thickness of three unit cells for the surface state calculation. The calculated surface band projections **(B)** without and **(C)** with the SOC effect. The drumhead surface states that originated from the topological crossing points are indicated by the black arrows.

In general, topological nodal ring or line states are characterized by drumhead surface states, which can be located either outside or inside the projected nodal ring or line. To examine the surface states associated with the Weyl nodal rings and the Dirac nodal lines in the BaAgBi material, we constructed a tight-binding Hamiltonian by projecting the Bloch states to atomic orbitals with maximally localized Wannier functions, as employed in the Wannier90 code. We built a slab model along the (001) surface and then calculated the corresponding surface states because the location of the nodal rings and lines are all parallel to this surface (Figure 3). Figure 6A illustrates the (001) surface slab model with only a thickness of three unit cells, but 20 layers were used for the calculations.

Figures 6B,C show the calculated topological surface states along the (001) surface projection for both the two Weyl nodal rings and the two Dirac nodal lines, respectively. It is worth noting that the bulk band structures are also overlayed on the surface projection, and they exhibit very good correspondence, particularly in the topological band crossing areas. As indicated by the black arrows in the figure, multiple drumhead surface states can be clearly observed, and they are all emitted from the nodal ring or nodal line crossing points. The surface states of the smaller Weyl nodal ring are well separated from the bulk band projection, while those of the larger ones are buried within the bulk states. The band projection with the SOC effect in Figure 6C is substantially more complicated than the clean band spectrum without the SOC effect in Figure 6B. However, the drumhead states are still noticeable. These surface states have a relatively large energy variation range and a relatively wide spatial distribution area, both of which are beneficial for further experimental detection. We highly encourage that ARPES experiments be performed to detect its surface states in the future.

## Conclusion

In this work, we used first-principles calculations to systematically study the topological properties of the ternary compound, BaAgBi. The calculated electronic band structures revealed the metallic feature of BaAgBi. Additionally, multiple topological band crossing points were discovered near the Fermi energy level. When the SOC effect was neglected, two Weyl nodal ring states were observed along the k_z_ = 0 plane. However, the topological states transformed into two Dirac nodal lines under the SOC effect, and their spatial distribution also shifted into the k_z_ = π plane. The surface projected states of BaAgBi along the (001) plane were calculated on a 20-layer surface slab model using a tight-binding Hamiltonian constructed from maximally localized Wannier functions. The Weyl nodal rings and the Dirac nodal lines both had a clear drumhead surface spectrum. Their spatial distributions and energy variations are very large, which can be beneficial for further experimental investigation.

## Data Availability

The original contributions presented in the study are included in the article/supplementary material, further inquiries can be directed to the corresponding author.
